# CD8^+^ T cells mediate the impact of gut dysbiosis and short-chain fatty acid deficiency on accelerated arthritis progression in collagen-induced arthritis mice

**DOI:** 10.3389/fimmu.2025.1702792

**Published:** 2025-11-14

**Authors:** Xiangcong Zhao, Yuhang Song, Wenpeng Zhao, Lili Shang, Chunxue Fan, Jianfang Xie

**Affiliations:** 1 Department of Rheumatology, The Second Hospital of Shanxi Medical University, Taiyuan, Shanxi, China; 2 Shanxi Key Laboratory for Immunomicroecology, The Second Hospital of Shanxi Medical University, Taiyuan, Shanxi, China

**Keywords:** gut dysbiosis, collagen-induced arthritis mice, short-chain fatty acid deficiencies, immune cells, immune phenotype

## Abstract

**Objective:**

This study aims to investigate the association between gut microbiota dysbiosis and phenotypic alterations in immune cells across multiple tissues in a collagen-induced arthritis (CIA) mouse model, and to elucidate the bidirectional regulatory mechanisms underlying the interaction between the gut microbiota and host immune responses.

**Methods:**

Twelve 6-8-week-old male DBA/1 mice were randomly assigned to either a collagen-induced arthritis (CIA) model group (n=6) or a normal (NOR) group (n=6). At the end of the experiment, feces, peripheral blood (PB), spleen (SP), intestinal segments, joint tissues and serum were collected. We employed an integrated analytical approach comprising fecal 16S ribosomal DNA gene sequencing, short-chain fatty acid (SCFA) metabolomics, flow cytometric detection of IgA-coated bacteria, immune phenotyping by flow cytometry, and cross-group network analysis to systematically evaluate gut microbial composition and host cellular immune profiles.

**Results:**

CIA mice developed polyarthritis, accompanied by a decrease in splenic T and NK cells and an increase in B cells. CD8^+^ T cells were significantly increased in mesenteric lymph nodes (MLN) and intestinal mucosa (IM). The gut microbiota exhibited reduced α-diversity, enriched Bacteroidetes, depleted Firmicutes, and decreased Lachnospiraceae_NK4A136_group. Fecal SCFA levels declined, while the proportion of IgA-coated bacteria increased. Functional prediction analysis indicated downregulation of microbial gene pathways associated with xylan decomposition, amino acid metabolism, and drug efflux, whereas pathways related to cell wall synthesis were upregulated. Cross-omics analysis confirmed significant correlations between these immune cells and specific bacterial genera.

**Conclusion:**

The reduction of SCFAs synthesis caused by gut microbiota dysregulation in CIA mice is related to the expansion of intestinal CD8^+^ T cells and may further promote the imbalance of T/B cells in the spleen; this gut microbiota -SCFA- CD8^+^ T cell axis may be involved in the occurrence and development of arthritis.

## 1 Introduction

Rheumatoid arthritis (RA) is a chronic systemic autoimmune disease characterized by progressive synovitis and joint destruction. Its associated irreversible bone erosion and significantly reduce patients’ quality of life, making it one of the most disabling medical conditions (1). The pathogenesis of RA is complex, involving T cells and B cells as central players driving abnormal activation of cellular and humoral immune responses, respectively. Previous studies have identified CD4^+^T cells as pivotal in RA pathogenesis, where abnormal hyperfunction of T helper 17 (Th17) cells and functional impairment in regulatory T cells (Tregs) can lead to sustained autoimmune responses and a breakdown of immune tolerance (2, 3). Recent evidence has also highlighted the important role of CD8^+^T cells in RA development. CD8^+^T cells in the peripheral blood and synovial fluid of RA patients are highly activated (3) and are capable of directly recognizing citrullinated fibrinogen and other autoantigens, and releasing perforin and granzyme to induce chondrocyte apoptosis and bone erosion (4). Abnormal activation of B cells leads to the production of autoantibodies such as rheumatoid factor (RF) and anti-cyclic citrullinated peptide antibody (ACPA), promoting osteoclast differentiation, inhibiting osteogenic function, and ultimately causing bone and joint destruction (5, 6). Additionally, neutrophils, macrophages, natural killer (NK) cells, dendritic cells, and other immune cells are involved in RA pathogenesis, forming an extremely complex immunopathological network together with T and B lymphocytes (7–9).

Gut microbiota dysbiosis is implicated in all stages of RA development, including the preclinical phase. It represents a key upstream factor driving abnormal immune responses in RA (10). As the largest microbial community in the human body, the gut and the host immune system maintain a dynamic symbiotic relationship, influencing, shaping, and regulating each other to sustain internal homeostasis. Disruption of the gut microbiota or compromise of immune defense can lead to aberrant autoimmune responses (11). Short-chain fatty acids (SCFAs), especially butyrate, provide energy for intestinal mucosal cells and play a central role in strengthening intercellular connectivity, maintaining epithelial integrity, and enhancing IgA secretion (12, 13). Studies have identified a microbial imbalance pattern—characterized by enrichment of Bacteroidota, depletion of Firmicutes, and reduction of butyrate-producing bacteria—that appears in high-risk RA populations years before autoantibodies such as ACPA become positive (14, 15). Thus, gut disturbance followed by reduced SCFA production is crucial in disrupting host intestinal immune balance and represents a key mechanism in RA pathogenesis.

In this study, we constructed a collagen-induced arthritis (CIA) mouse model, collected feces, peripheral blood, spleen, intestine, joints, and other tissues, and systematically analyzed the complex relationship between gut microbiota dysbiosis and immune homeostasis using 16S rDNA sequencing, SCFA metabolomics, IgA-coated bacteria detection, and flow cytometry. Our findings provide a theoretical foundation for further elucidating the pathogenesis of RA.

## 2 Materials and methods

### 2.1 CIA model preparation

All animal experiments were approved by the Institutional Animal Care and Use Committee of the Second Hospital of Shanxi Medical University and were performed in accordance with the relevant guidelines and regulations. Twelve male DBA/1 mice (6–8 weeks old) were purchased and acclimatized in a specific pathogen-free environment for 2 weeks. Six mice were randomly assigned to the collagen-induced arthritis (CIA) model group, while the remaining six served as the normal control group (NOR). The CIA model was established according to a modified protocol from Miyoshi et al. (16). Briefly, a 2 mg/mL solution of bovine type II collagen (CII; Chondrex Inc., USA) was emulsified at a 1:1 ratio with either 4 mg/mL complete Freund’s adjuvant (CFA; Chondrex Inc.) or 2 mg/mL incomplete Freund’s adjuvant (IFA; Chondrex Inc.). On day 1 of immunization, each mouse received a 100 μL injection of the CII/CFA emulsion subcutaneously at the base of the tail. A booster immunization was administered on day 21 by injecting 100 μL of the CII/IFA emulsion subcutaneously at the base of the tail. Each mouse received a total of 200 µg of type II collagen in two doses. Arthritis severity was assessed weekly using a rheumatoid arthritis joint damage (RAAD) scoring system: 0 = no signs of erythema or swelling; 1 = erythema and mild swelling confined to the tarsus or ankle joint; 2 = erythema and mild swelling extending from the ankle to the tarsus; 3 = erythema and moderate swelling extending from the ankle to the metatarsal joints; 4 = erythema and severe swelling involving the ankle, foot, and digits, or apparent joint stiffness.

### 2.2 Anesthesia, euthanasia, and handling of mice

In this experiment, inhalation anesthesia was performed on mice using a small animal anesthesia machine (Shenzhen RWD TAIJI Series), with the experimental anesthetic being Isoflurane (Batch No.: 2024081901).The inflammatory peak occurred approximately 10 weeks after the initial immunization and all mice were anesthetized and euthanized at the 11th week after the initial immunization. The anesthesia concentration is 3.0%, and the euthanasia concentration is 5.0%.The detailed anesthesia process is included in the supplementary.

### 2.3 Histopathological analysis of peripheral joints

The right hind limb was dissected and fixed in 10% neutral buffered formalin, followed by decalcification, paraffin embedding, and sectioning. Standard histological staining techniques, including hematoxylin and eosin (H&E), safranin-O, and toluidine blue, were employed to evaluate pathological features such as synovial hyperplasia, inflammatory cell infiltration, pannus formation, and bone erosion.

### 2.4 Analysis of immune phenotypes in peripheral blood, spleen, and intestinal tissues

#### 2.4.1 Pretreatment of peripheral blood, spleen, and intestinal tissue

Peripheral blood (PB) was collected following euthanasia. Serum was separated by centrifugation and stored at –80°C for subsequent analysis. Spleens (SP) and mesenteric lymph nodes (MLN) were collected aseptically, with an average of 5 mesenteric lymph nodes per mouse. Peyer’s patches (PPs) were collected from the ileal segment starting at the ileocecal junction and extending approximately 8–10 centimeters toward the oral direction, with approximately 8 PPs per mouse. Tissues including SP, MLN, and PPs were gently pressed through a 100 μm cell strainer to generate single-cell suspensions. Target cells were subsequently isolated using Percoll density gradient centrifugation.

Intestinal mucosal (IM) tissue was collected from the jejunum and ileum of mice, thoroughly washed with phosphate-buffered saline (PBS), cut into fragments of approximately 5 mm, and repeatedly flushed with pre-warmed Hanks’ Balanced Salt Solution (HBSS) at 37°C. The tissue pieces were transferred to a 50 mL centrifuge tube and digested with 10 mL of EDTA-containing digestion solution with continuous shaking at 37°C for 10 minutes. The digest was filtered through sterile gauze, and the retained tissue fragments were rinsed 3–4 times with fresh EDTA digestion solution. The resulting cell suspension was sequentially passed through 100 μm and 40 μm cell strainers, and centrifuged at 600 × g for 6 minutes. The supernatant was carefully discarded, and the cells were resuspended in 1 mL of FACS buffer. Finally, the cells were further purified by a Percoll density gradient centrifugation.

#### 2.4.2 Detection of lymphocytes and NK cells

The following immune cell populations were detected: total T cells (CD45^+^CD3^+^), total B cells (CD220^+^CD3^-^), natural killer (NK) cells (CD3^-^CD335^+^), CD4^+^ T cells (CD45^+^CD3^+^CD4^+^), and CD8^+^ T cells (CD45^+^CD3^+^CD8^+^). The following fluorescently conjugated antibodies were used: anti-CD45 (BV510, Rat Anti-Mouse, BD Biosciences), anti-CD45 (PerCP-Cy5.5, Rat Anti-Mouse, BD Biosciences), anti-CD3ϵ (FITC, Hamster Anti-Mouse, BD Biosciences), anti-CD4 (PE-Cy™7, Rat Anti-Mouse, BD Biosciences), anti-CD8a (APC-Cy™7, Rat Anti-Mouse, BD Biosciences), anti-B220 (APC, Rat Anti-Mouse, BD Biosciences), and anti-CD335 (PE, Rat Anti-Mouse, BD Biosciences).

For peripheral blood samples, 50 μL of EDTA-anticoagulated whole blood was aliquoted into flow cytometry tubes. The appropriate antibodies were added according to the manufacturer’s instructions, mixed gently, and incubated at room temperature (20-25°C) for 15 minutes. Then, 450 μL of 1× lysing solution was added, and the samples were incubated in the dark at room temperature for 15–20 minutes. For SP, MLN, and PPs samples, single-cell suspensions were prepared and adjusted to a concentration of 1×10^6^ cells per tube for surface staining. The staining procedure was identical to that used for peripheral blood samples. All samples were analyzed using a BD FACSLyric flow cytometer (Becton Dickinson). The results in the text, figures, and tables represented the percentage of cells.

#### 2.4.3 Detection of dendritic cells

The following dendritic cell (DC) subsets were identified and analyzed: total DCs (CD45^+^I-A/I-E^+^), classical DCs (cDCs; CD45^+^I-A/I-E^+^CD11c^+^), plasmacytoid DCs (pDCs; CD45^+^I-A/I-E^+^CD317^+^), CD8α^+^ cDC1 (CD45^+^I-A/I-E^+^CD11c^+^CD8α^+^), CD103^+^ cDC1 (CD45^+^I-A/I-E^+^CD11c^+^CD103^+^), and cDC2 (CD45^+^I-A/I-E^+^CD11b^+^). The following fluorescently conjugated antibodies were used for staining: anti-CD45 (BV510, Rat Anti-Mouse, BD Biosciences), anti-I-A/I-E (PerCP-Cy™5.5, Rat Anti-Mouse, BD Biosciences), anti-CD8a (APC-Cy™7, Rat Anti-Mouse, BD Biosciences), anti-CD11b (PE-Cy™7, Rat Anti-Mouse, BD Biosciences), anti-CD317 (APC, Rat Anti-Mouse, eBioscience), anti-CD11c (BV421, Rat Anti-Mouse, eBioscience),and anti-CD103(Brilliant Violet 605™ anti-mouse CD103 Antibody,Biolegend).The staining procedure was identical to that described in section 2.3.2. The results in the text, figures, and tables represented the percentage of cells.

### 2.5 Enzyme-linked immunosorbent assay

Serum levels of type II collagen-specific IgG antibodies were measured using an ELISA kit (Chondrex, Inc.) according to the manufacturer’s instructions. Absorbance was measured at 450 nm on a TECAN Sunrise microplate reader.

### 2.6 Detection of intestinal microbiota

#### 2.6.1 Metabolomic analysis

A total of 30 mg of fecal sample was homogenized in 300 μL of ultrapure water by vortexing for 3 minutes, incubated at 4°C for 10 minutes, and then centrifuged at 12,000 × g for 10 minutes. Then, 50 μL of the supernatant was mixed with 80 μL of methanol, 4 μL of a 0.2 mg/mL internal standard solution, and 6 μL of 0.5% (v/v) sulfuric acid. The mixture was filtered through a 0.22 μm membrane prior to analysis. A standard mixture of short-chain fatty acids (SCFAs)—including acetic acid (AA), propionic acid (PA), isobutyric acid (IBA), butyric acid (BA), isovaleric acid (IVA), and valeric acid (VA)—was prepared by dissolving 10 μL of each standard in 50% (v/v) methanol and diluting to a final volume of 1 mL, yielding a concentration of 10 μL/mL for each SCFA. An internal standard stock solution was prepared by dissolving 10 μL of D9-isovaleric acid (D9-IVA) in 50% methanol and diluting to 1 mL. Quantitative analysis of SCFAs was performed using a GC2030 gas chromatograph coupled with an MS-TQ8050 mass spectrometer (Shimadzu, Japan) equipped with an SH-PolarCap capillary column (30 m × 0.25 mm × 0.25 μm). The GC conditions were as follows: high-purity helium was used as the carrier gas at a flow rate of 1.0 mL/min; the split ratio was set to 20:1; the injection volume was 0.5 μL; and the inlet temperature was maintained at 175°C. The temperature program consisted of an initial hold at 100°C for 1 minute, followed by an increase to 190°C at a rate of 12°C/min, then a ramp to 220°C at 30°C/min, and a final hold at 220°C for 3 minutes.

#### 2.6.2 Flow cytometry

A total of 20 mg of fecal sample was homogenized in phosphate-buffered saline (PBS) at a concentration of 25 mg/mL using vortex mixing and ultrasonic dissolution. The homogenate was centrifuged at 8000 × g for 5 minutes, and the supernatant was collected. The pellet was resuspended in 1 mL of 5 mM N-acetylcysteine solution, incubated for 5 minutes, and centrifuged again at 8000 × g for 5 minutes. The supernatant was discarded, and the pellet was washed twice with PBS, then passed through a 70 μm cell strainer. The filtrate was resuspended in 1 mL PBS and centrifuged to collect the sediment. The fecal sediment was suspended in 20% fetal bovine serum (FBS) and incubated for 20 minutes. After two additional washes with PBS, the sample was transferred to a flow cytometry tube and stained with DAPI and an IgA-specific antibody(anti-mouse IgA PE,eBioscience). Following a 30-minute incubation, the sample was washed, diluted to a final volume of 1 mL, and filtered through a 35 μm strainer. Analysis was performed using a BD LSRFortessa flow cytometer (Becton Dickinson).

#### 2.6.3 16S rDNA gene sequencing

DNA concentration and purity were assessed by agarose gel electrophoresis using an Agilent 5400 system. The V3–V4 hypervariable region of the 16S rRNA gene was amplified with the universal primers 341F (5′-CCTAYGGGRBGCASCAG-3′) and 806R (5′-GGACTACNNGGGTATCTAAT-3′). Each 25 μL PCR reaction contained 15 μL of Phusion High-Fidelity PCR Master Mix (New England Biolabs), 0.2 μM of each primer, and 10 ng of genomic DNA template. The thermal cycling conditions were as follows: initial denaturation at 98°C for 1 min; 30 cycles of denaturation at 98°C for 10 s, annealing at 50°C for 30 s, and extension at 72°C for 30 s; followed by a final extension at 72°C for 5 min. The resulting PCR products were purified using magnetic beads, and target amplicons were excised and recovered after electrophoresis. Sequencing libraries were prepared and subjected to Illumina sequencing following quantification with Qubit fluorometric measurement and quantitative PCR (qPCR).

### 2.7 Bioinformatics analysis

All effective tags were clustered into operational taxonomic units (OTUs) at 97% similarity using the UPARSE algorithm (v7.0.1001). The most abundant sequence within each OTU was selected as the representative sequence and taxonomically annotated against the SILVA 138.1 database. To maintain consistency with previous studies, this study used QIIME (v1.9.1) and PICRUSt (v1.1.4) for microbiome analysis, versions that have been validated in our laboratory for similar data processing. Alpha diversity indices, including Observed OTUs, Chao1, Shannon, Simpson, ACE, Goods coverage, and PD whole tree, were calculated using QIIME. Rarefaction curves were generated with R software (v4.0.3). Beta diversity was assessed based on both weighted and unweighted UniFrac distances, which were computed within QIIME. MetagenomeSeq was employed to identify differentially abundant species. Biomarkers were identified using linear discriminant analysis effect size (LEfSe), with significance thresholds set at LDA score > 4 and p < 0.05. Metagenomic functional potential was predicted with PICRUSt, and ecological functional annotations were inferred using FAPROTAX. Principal coordinate analysis (PCoA) was performed with the ade4 and ggplot2 packages in R. Spearman correlation analysis, canonical correspondence analysis (CCA), redundancy analysis (RDA), and distance-based redundancy analysis (dbRDA) were applied to evaluate correlations between environmental factors and microbial abundance. All statistical and visual analyses were conducted in the R environment.

### 2.8 Statistical analysis

Statistical analyses were performed using SPSS version 27.0 (SPSS Inc., Chicago, IL, USA). A p-value of less than 0.05 was considered statistically significant. The normality of data distribution was assessed using the Shapiro-Wilk test. Homogeneity of variances was assessed using Levene’s test. Data with normal distribution and homogeneity of variance are presented as mean ± standard deviation. Comparisons between groups were conducted using independent sample t-tests. Non-normally distributed data were analyzed using the Wilcoxon rank-sum test. Correlation analyses were performed using either Pearson or Spearman methods, as appropriate.

## 3 Results

### 3.1 Arthritis manifestations and pathological grading of CIA mice

**Figure 1:**
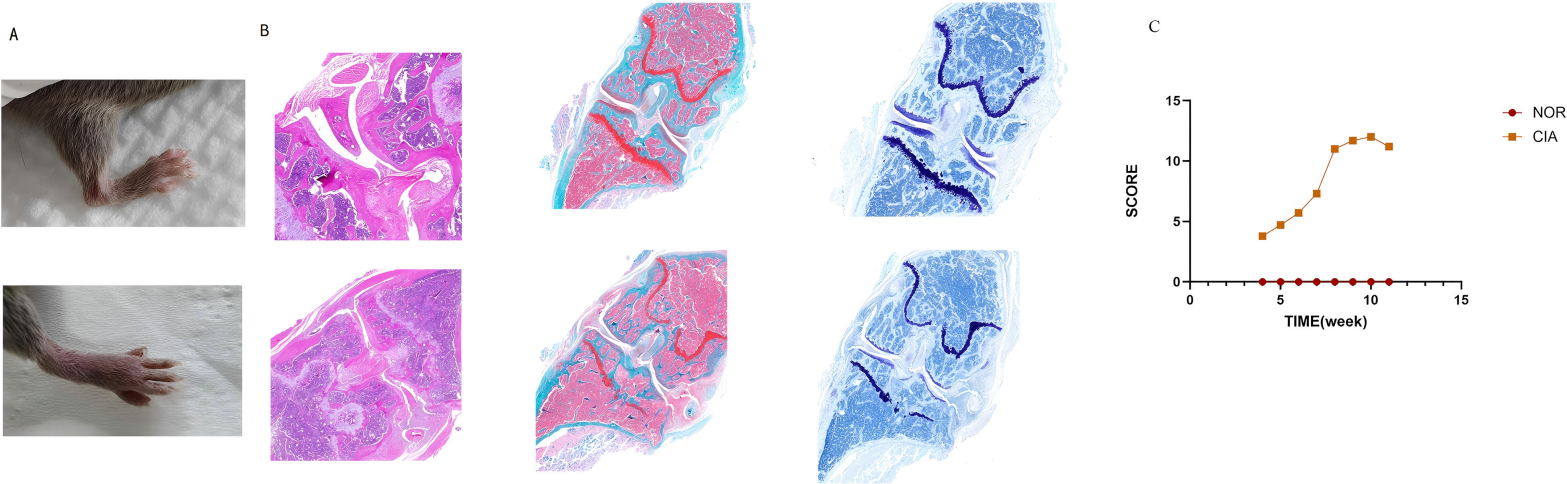
CIA mice exhibited marked arthritic manifestations. **(A)** Redness and swelling in the ankle and interphalangeal joints. **(B)** In CIA mice, inflammatory cell infiltration and pannus formation resulted in articular cartilage erosion, marked proteoglycan loss, and chondrocyte shrinkage, culminating in the destruction of joint architecture. **(C)** Dynamic changes of the arthritis index (AI) in mouse joints. (There are 6 NOR mice and 6 CIA mice.)

Following initial immunization, CIA mice exhibited reduced feeding and activity, slight hair loss, and ulcer formation at injection sites, which healed within approximately one week. Subsequently, joints showed symmetrical redness and swelling, initially affecting the hind limbs and progressively involving the knees, ankles, wrists, and interphalangeal joints, with symptoms worsening over time. By the 10th week, the joint score reached its maximum. (**Supplementary Table 1**) Ankylosis, deformity, and functional impairment developed around 6–8 weeks after immunization. The inflammatory peak occurred approximately 10 weeks after the initial immunization, followed by progression into the chronic phase (**Figures 1A, C**).

H&E staining showed that in the NOR group, synovial cells were arranged neatly, with no observed inflammatory cell infiltration; the joint cavity structure was clear, and the cartilage surface was smooth. In contrast, the CIA group exhibited significant synovial hyperplasia, disorganized cell arrangement, extensive inflammatory cell infiltration, and synovitis. Synovial tissue invasion into cartilage and bone was also observed, leading to noticeable bone erosion and joint structure destruction. Safranin O and Toluidine Blue staining further indicated that in the CIA group, the cartilage surface was damaged, proteoglycans were largely lost, and chondrocyte shrinkage was observed (**Figure 1B**). ELISA detection of serum type II collagen IgG antibody levels revealed that the mean antibody level in the CIA group was 7.56 ± 1.4 U/ml, whereas in the NOR group, levels were below the detection limit; the difference between the groups was statistically significant. These results indicate that the CIA model was successfully established.

### 3.2 Immune phenotype changes in CIA mice

#### 3.2.1 Phenotypic changes of immune cells in peripheral blood and spleen

**Figure 2:**
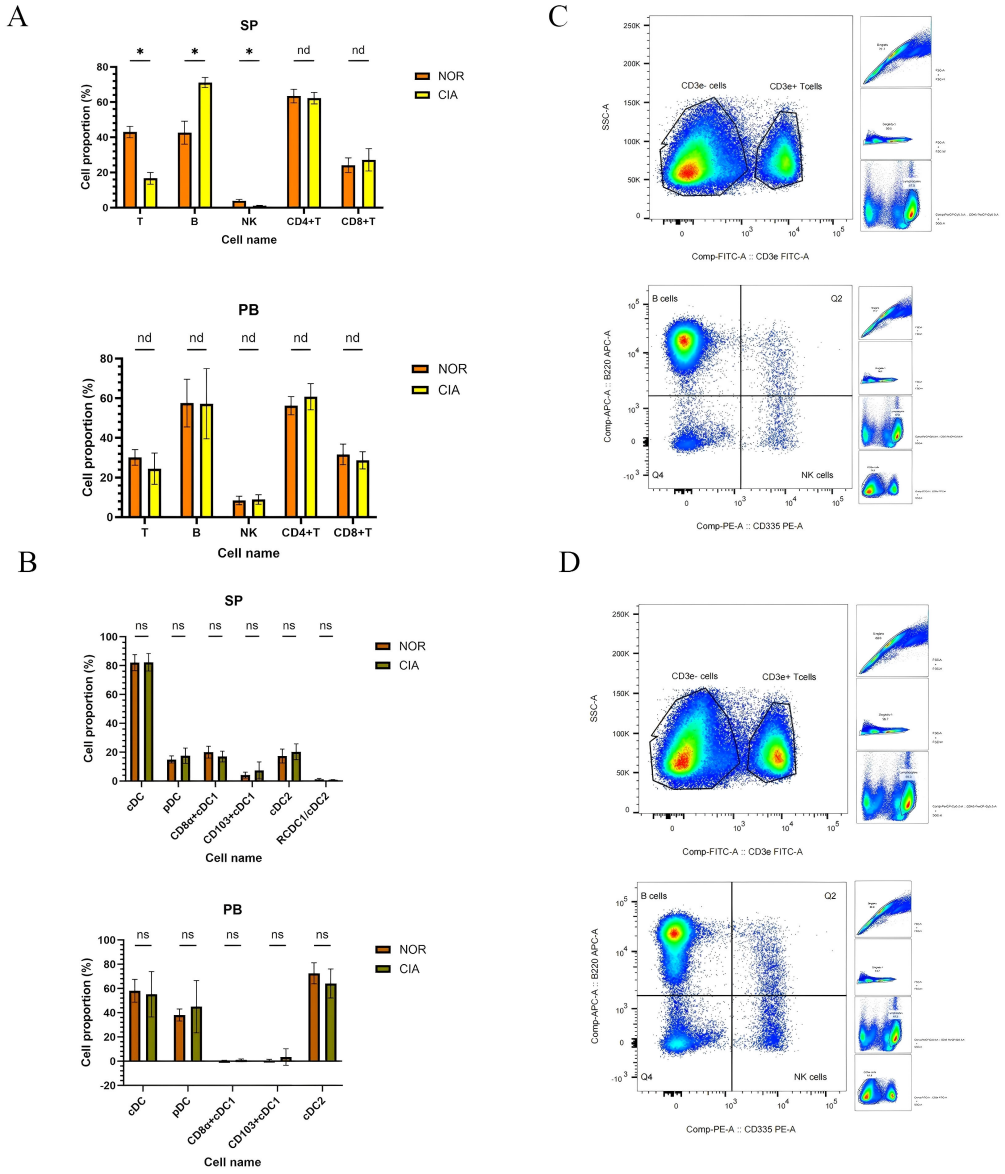
In CIA mice, splenic total T and NK cell levels were reduced, whereas B-cell levels were elevated; no significant differences were detected in dendritic cell (DC) subsets in peripheral blood (PB) or spleen (SP). **(A)** Immune cell phenotype changes in SP and PB. **(B)** Changes in DCs subsets between SP and PB. x-axes indicate individual cell populations; y-axes indicate the percentage of each cell population. (*P < 0.05). **(C)** Representative flow-cytometric dot plots of T, B, and NK cells for the NOR group (upper) and the CIA group (lower). nd, No Difference; ns, Not Significant.

No significant differences were observed in the levels of total T cells, B cells, natural killer (NK) cells, CD4^+^ T cells, or CD8^+^ T cells in Peripheral blood (PB) of CIA mice compared to the NOR group. In Spleen (SP), however, total T cells (*p* < 0.05) and NK cells (*p* < 0.05) were significantly decreased, while B cells were significantly increased (*p* < 0.05). The proportions of CD4^+^ T cells and CD8^+^ T cells did not change significantly.(**Figure 2A**). (For specific figures, see the **Supplementary Tables 2A, B**).

No significant differences were detected in dendritic cell (DC) subsets between the two groups in peripheral blood (PB) or spleen (SP). (**Supplementary Tables 2C, D**) However, distinct distribution patterns were noted: CD11b^+^ cDC2 was the dominant subset in PB, whereas both CD8α^+^ cDC1 and CD11b^+^ cDC2 were present in SP, although the ratio of CD8α^+^ cDC1 to CD11b^+^ cDC2 showed a decreasing trend (**Figure 2B**).

#### 3.2.2 Phenotypic changes of local intestinal immune cells

**Figure 3:**
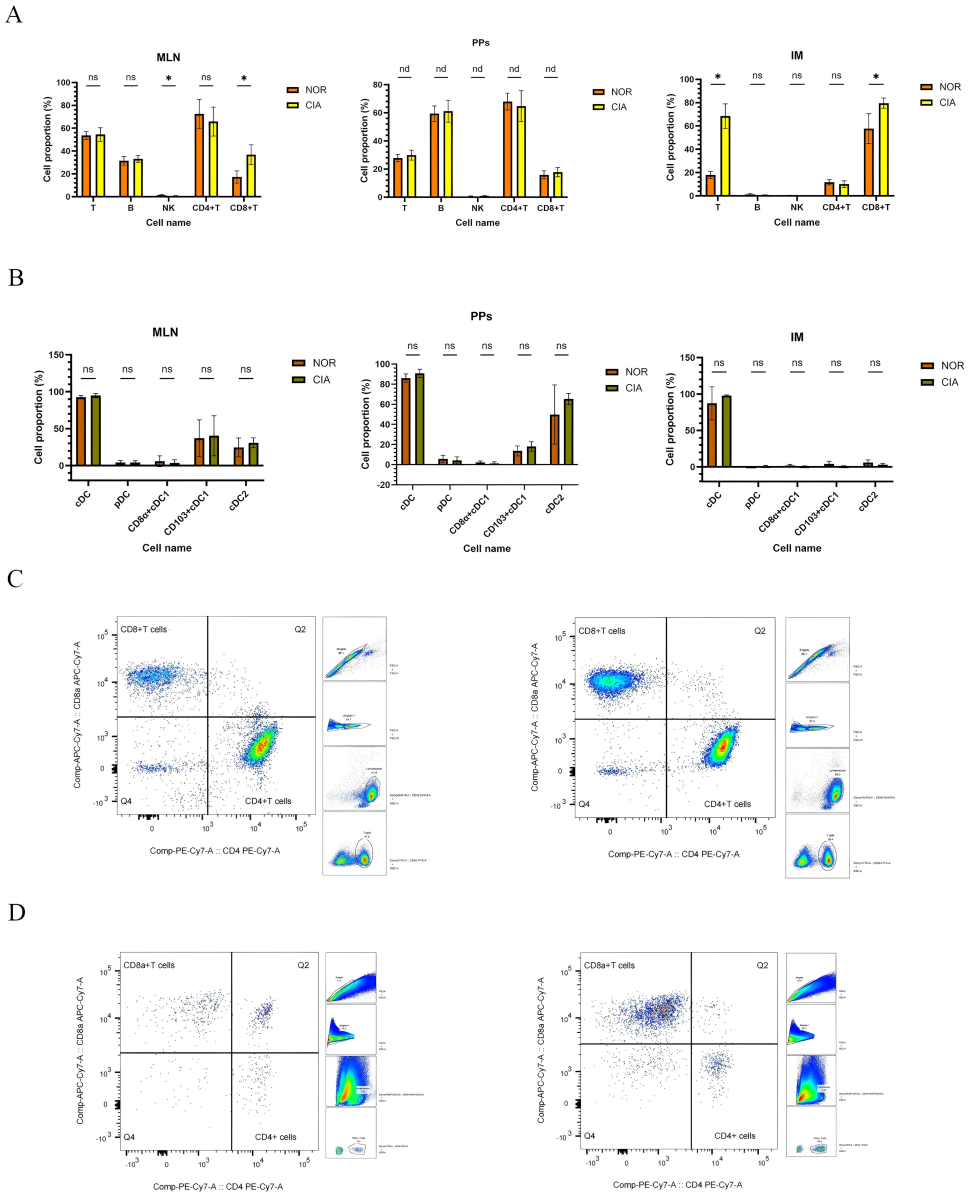
Immune-cell profiles in mesenteric lymph nodes (MLN), Peyer’s patches (PPs), and intestinal mucosa (IM), with representative flow cytometry plots of CD4^+^ and CD8^+^ T cells. **(A)** Comparison of immune cell populations in MLN, PPs, and IM (data shown separately for each tissue). **(B)** Changes in dendritic cell (DC) subsets in MLN, PPs, and IM. (*P < 0.05). **(C)** Representative flow cytometry plots of CD4^+^ and CD8^+^ T cells in MLN. **(D)** Representative flow cytometry plots of CD4^+^ and CD8^+^ T cells in IM. NOR group (left) and CIA group (right). nd, No Difference; ns, Not Significant.

Significant alterations were observed in local intestinal immune cell populations in CIA mice. In mesenteric lymph nodes (MLN), NK cell levels were significantly decreased (*P* = 0.01), while CD8^+^T cells were significantly increased (*P* = 0.0008). CD4^+^T cells showed a decreasing trend, though total T and B cell levels showed no significant differences. (**Figures 3A, C**) (**Supplementary Table 3A**) No significant differences were detected in immune cell populations in Peyer’s patches (PPs); (**Supplementary Table 3B**) however, T cells, B cells, NK cells, and CD8^+^T cells showed an increasing trend, while CD4^+^T cells showed a decreasing trend (**Figure 3A**). In IM, T cells (*p* < 0.05) and CD8^+^T cells (*p* = 0.003) were significantly increased, whereas B cells, NK cells, and CD4^+^T cells showed no significant changes (**Figures 3A, D**) (**Supplementary Table 3C**).

No significant differences were found in dendritic cell (DC) subsets across intestinal tissues. CD103^+^ cDC1 and CD11b^+^ cDC2 were identified as the predominant DC subsets in both MLN and PPs (**Figure 3B**) (For specific figures, see the **Supplementary Tables 4A-C**).

### 3.3 Gut microbiota changes in CIA mice

#### 3.3.1 Changes in gut microbiota diversity

**Figure 4:**
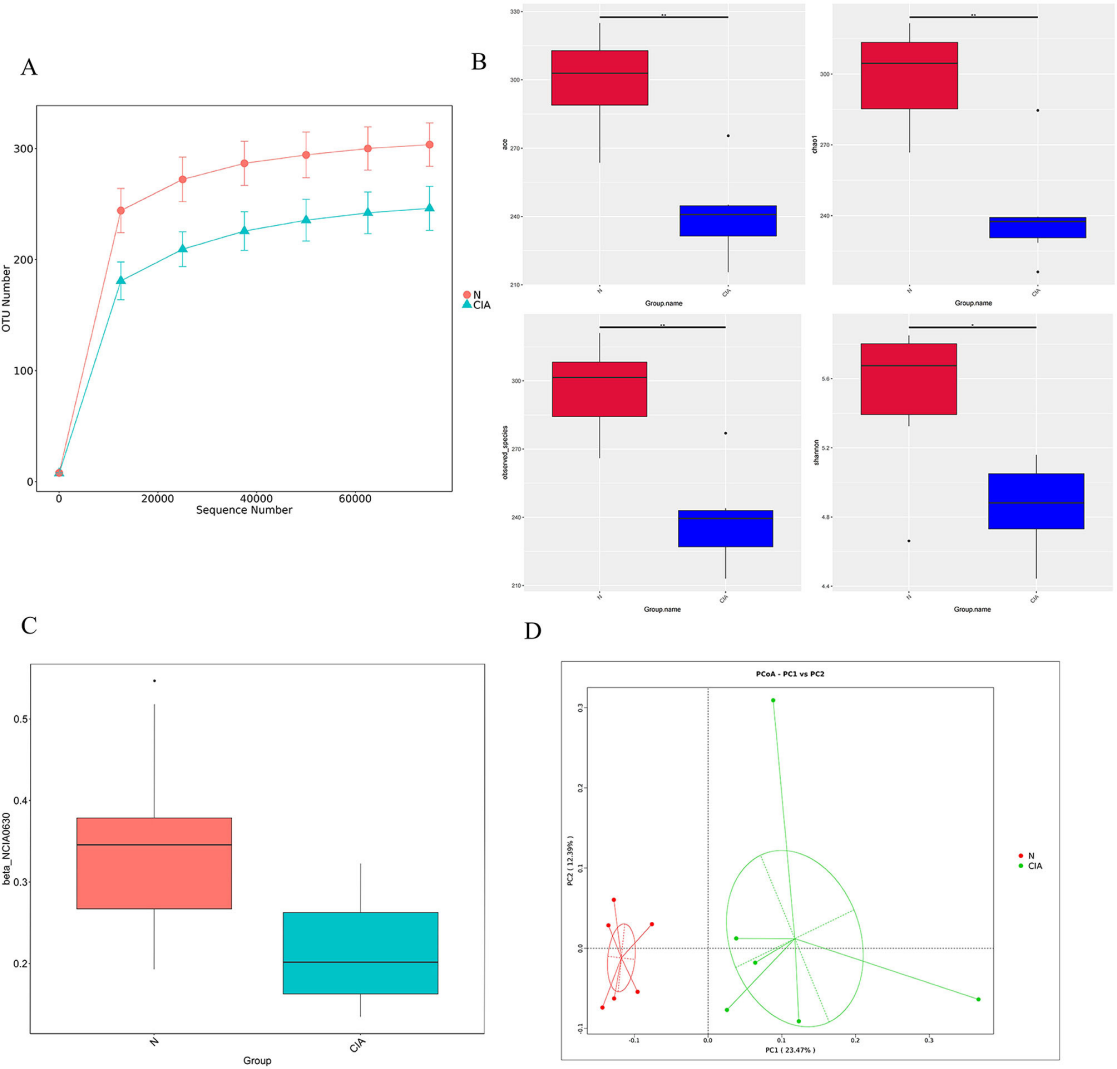
The gut microbiota of CIA mice differed markedly from that of normal mice. **(A)** Rarefaction curves for each sample. **(B)** Alpha-diversity indices (ACE, Chao1, observed species, Shannon) in the NOR and CIA groups (*P < 0.05, **P < 0.01). **(C)** Weighted UniFrac PCoA analysis. **(D)** Beta-diversity analysis based on binary Jaccard distances. Colors indicate group membership; the distance between any two points reflects the degree of microbial community dissimilarity—greater distances indicate lower similarity and larger differences between samples.

Microbiome sequencing yielded 672 operational taxonomic units (OTUs) following clustering with the UPARSE algorithm. Species accumulation curves confirmed that sequencing depth was sufficient for analysis. (**Figure 4A**) Alpha-diversity was significantly lower in the CIA group compared to the NOR group (ace, *p* < 0.001; Chao1, *p* = 0.008; observed species, *p* = 0.004; Shannon, *p* = 0.041) (**Figure 4B**). Beta-diversity analysis revealed significant separation between the groups, as assessed by both weighted UniFrac (*p* = 0.0002, Wilcoxon rank-sum test; **Figure 4C**) and binary Jaccard distances (**Figure 4D**). This confirms substantial alterations in the composition and structure of the gut microbiota in CIA mice.

#### 3.3.2 Analysis of differences in gut microbiota composition

**Figure 5:**
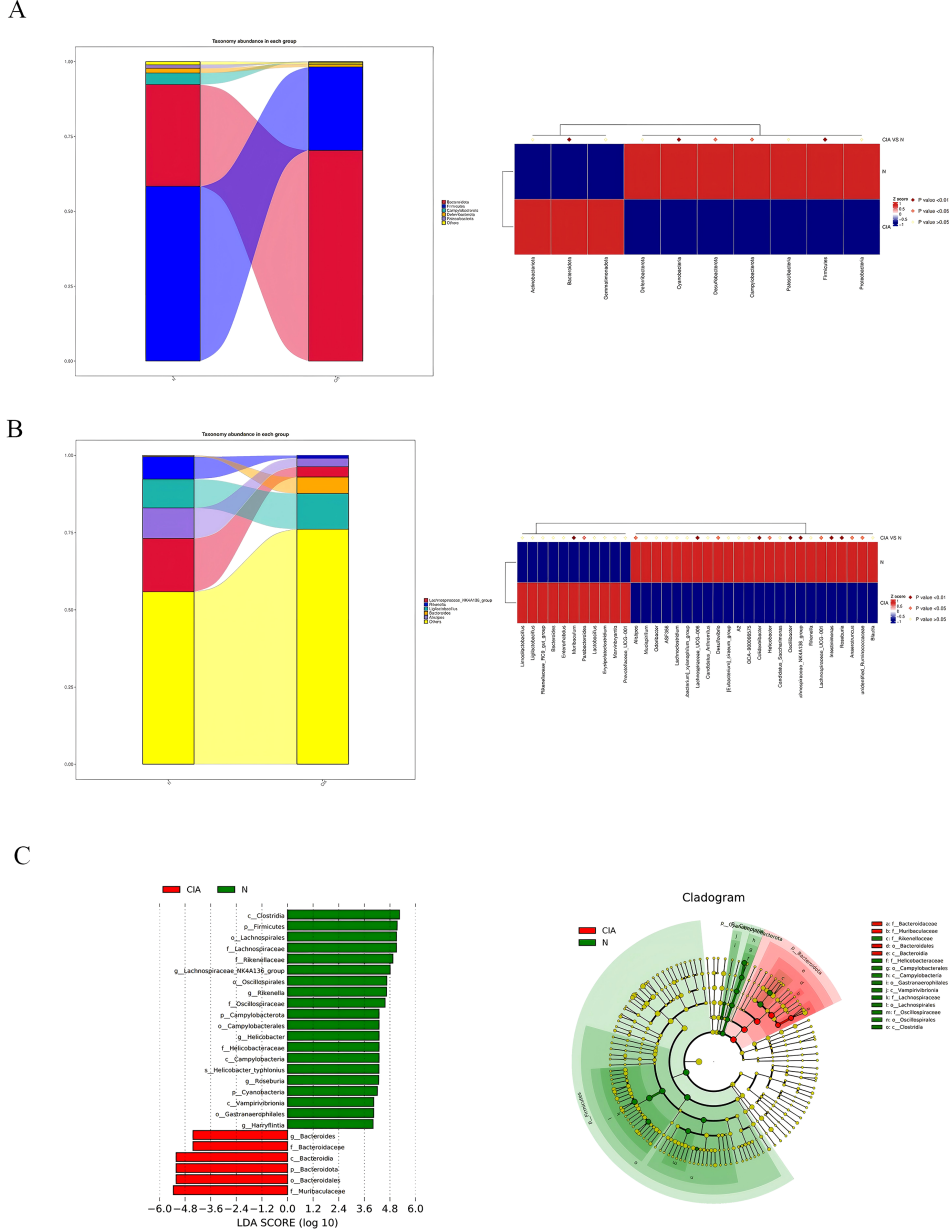
Changes in the composition and relative abundance of gut microbiota in CIA mice. **(A)** Phylum-level gut microbiota abundance in the NOR group and CIA group. **(B)** Genus-level gut microbiota abundance in the NOR group and CIA group. Heatmap: the redder the color, the higher the abundance; the bluer the color, the lower the abundance. **(C)** LDA score histogram (each bar represents a species, with bar length corresponding to the LDA score; the longer the bar, the greater the difference). Bar colors indicate group association. Phylogenetic circular diagram: circles from the inside out correspond to taxonomic levels. Lines indicate phylogenetic relationships. Circles represent taxonomic units; yellow circles indicate no significant differences between groups; other colors indicate higher abundance in the specified group.

Significant differences in microbial composition were observed between the two groups. At the phylum level, Bacteroidota was significantly enriched in the CIA group (*p* < 0.01), while Firmicutes (*p* < 0.01) and Campylobacterota (*p* < 0.05) showed significant reduction. (**Figure 5A**) At the genus level, *Parabacteroides* (*p* < 0.05) and *Muribaculum* (*p* < 0.01) were significantly increased, whereas key butyrate-producing bacteria including *Lachnospiraceae_NK4A136_group* (*p* < 0.01),Oscillibacter (*p* < 0.01) and *Roseburia* (*p* < 0.01) were significantly decreased. (**Figure 5B**) LEfSe analysis identified 26 characteristic bacterial groups (LDA > 4, *P* < 0.05). The CIA group was characterized by a dominance of Bacteroides, whereas the NOR group showed enrichment of Rikenella, Lachnospiraceae_NK4A136_group, and Roseburia. (**Figure 5C**) These results indicate that compositional differences, particularly the depletion of butyrate-producing bacteria, may contribute to CIA pathogenesis.

#### 3.3.3 Functional prediction of gut microbiota

**Figure 6:**
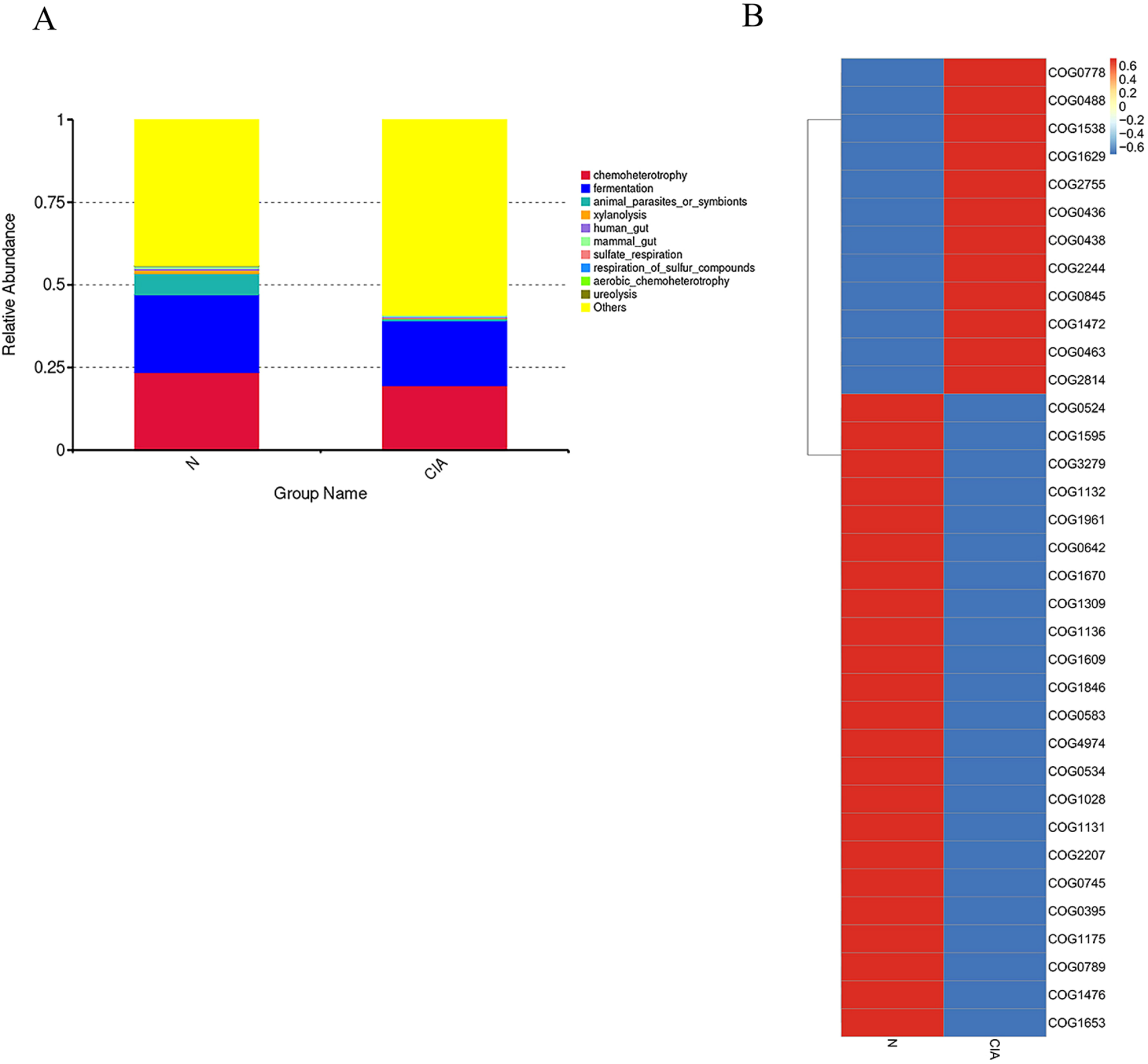
Differential functional prediction analysis of the gut microbiota. **(A)** Top 10 most significantly enriched pathways annotated by FAPROTAX. **(B)** Heatmap of COG functional pathways.

FAPROTAX prediction further indicated a reduced functional potential for fermentation, chemotrophy, animal parasitism/symbiosis, and xylanolysis in the CIA group. (**Figure 6A**) Analysis of the top 35 most differentially abundant pathways based on the COG database revealed significant functional alterations in the CIA group(**Figure 6B**). Pathways associated with O-antigen and phosphoteichoic acid output membrane proteins, as well as periplasmic β-glucosidase, were significantly upregulated. In contrast, pathways involving XerD site-specific recombinase, AraC-type DNA-binding protein, outer membrane protein TolC, aspartate/methionine/tyrosine aminotransferase, ATPase components of ABC-type multidrug transport systems, and transmembrane enzymes were significantly downregulated. These results suggest that the gut in CIA mice undergoes adaptive functional remodeling.

#### 3.3.4 Analysis of IgA coating of gut microbiota

**Figure 7:**
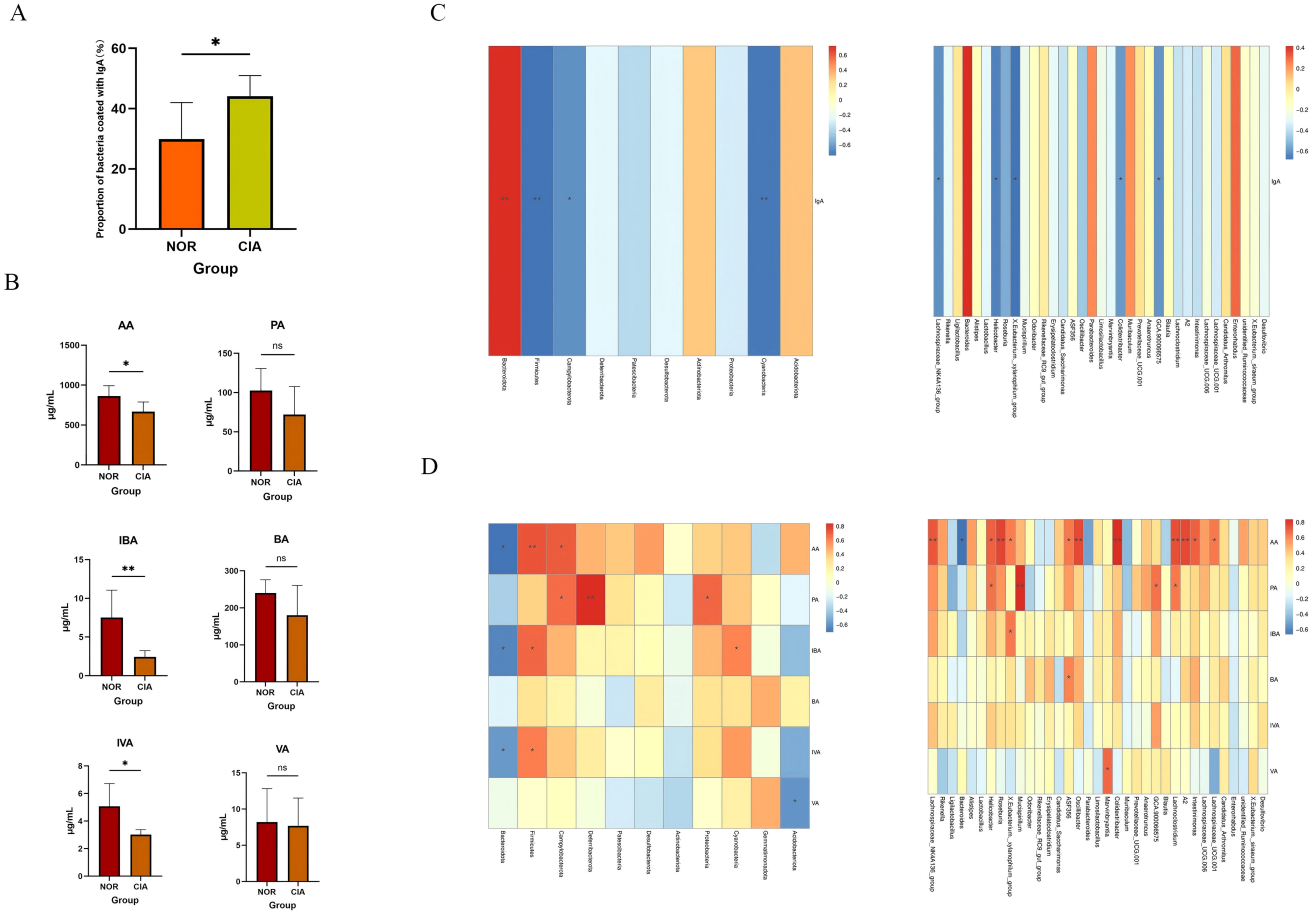
**(A)** Proportion of IgA-coated bacteria. **(B)** Concentrations of short-chain fatty acids (SCFAs). **(C)** Correlations of IgA-coated bacteria at the phylum and genus levels. **(D)** Correlations of fecal SCFAs at the phylum and genus levels. Red intensity denotes stronger positive correlations; blue intensity denotes stronger negative correlations (*P < 0.05, **P < 0.01). ns, Not Significant.

Flow cytometry results showed that, compared with the control group, the proportion of IgA-coated bacteria in the gut of CIA mice was significantly increased (p < 0.05) (**Figure 7A**). At the phylum level, the proportion of IgA-coated bacteria was positively correlated with the relative abundance of Bacteroidetes, and negatively correlated with the relative abundance of Firmicutes and Cyanobacteria, as well as with the order Campylobacterales. At the genus level, this proportion was significantly negatively correlated with Lachnospiraceae_NK4A136_group and Helicobacter (**Figure 7C**). Further correlation analysis with the percentages of B cells in the spleen (SP), peripheral blood (PB), mesenteric lymph nodes (MLN), Peyer’s patches (PPs), and intestinal mucosa (IM) revealed no significant associations. (**Supplementary Table 6**) These results indicate that the IgA coating status of the gut microbiota in CIA mice is closely linked to the local mucosal immune system.

#### 3.3.5 Metabolomic analysis of gut microbiota

The levels of short-chain fatty acids (SCFAs), including acetic acid (AA) (*p* < 0.05), isobutyric acid (IBA) (*p* < 0.05), and isovaleric acid (IVA) (*p* < 0.05), were significantly reduced in the CIA group compared to the NOR group. In contrast, propionic acid (PA), butyric acid (BA), and valeric acid (VA) also exhibited decreasing trends, though these changes were not statistically significant. (**Figure 7B**) (**Supplementary Table 5**) Spearman correlation analysis indicated that AA, IBA, and IVA levels were positively correlated with the abundance of Firmicutes and negatively correlated with Bacteroidota. Furthermore, AA levels were positively correlated with butyrate-producing bacteria (e.g., *Lachnospiraceae_NK4A136_group* and *Roseburia*) and negatively correlated with *Bacteroides* (**Figure 7D**).

### 3.4 Cross-group network analysis of gut microbiota-immunity-disease

#### 3.4.1 Correlation between OTU abundance and immune cell phenotype

**Figure 8:**
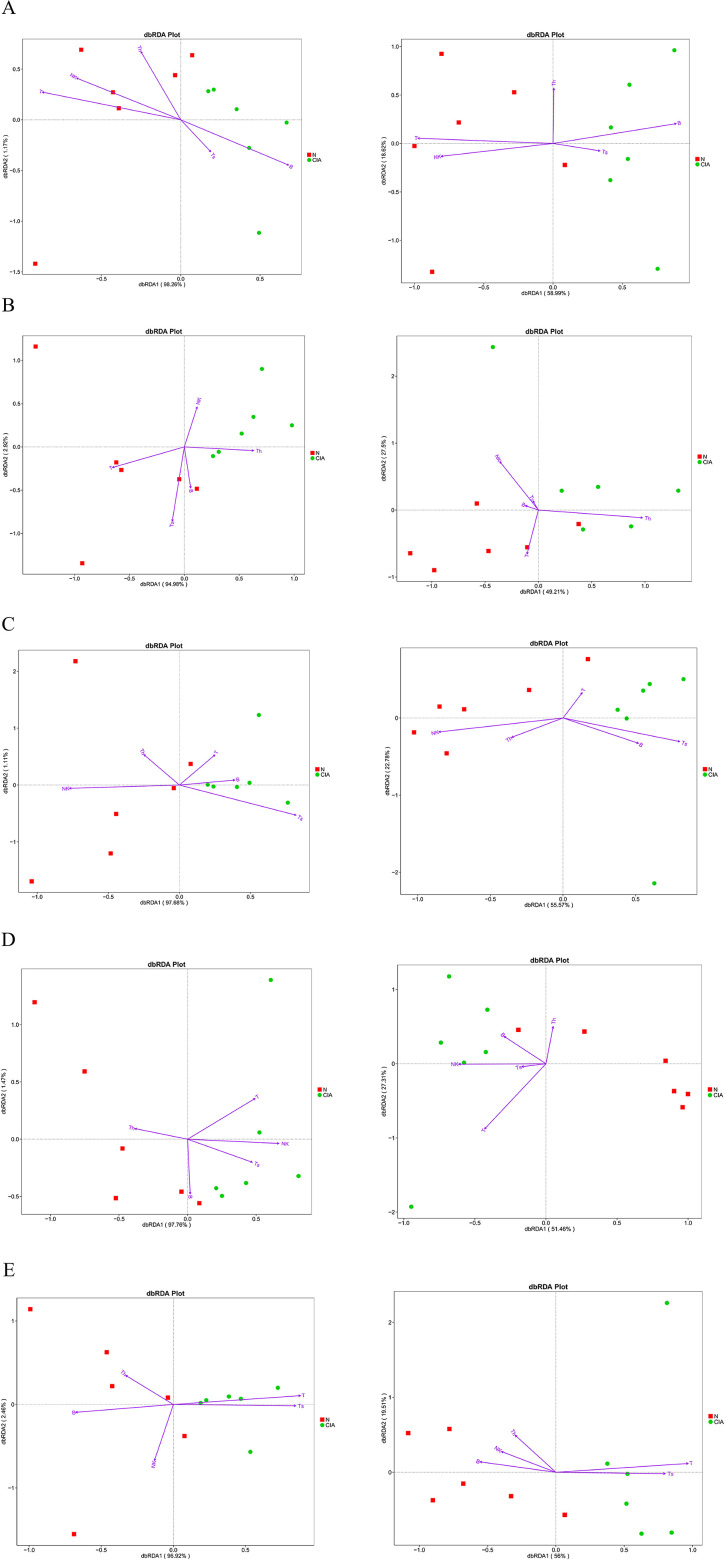
Distance-based redundancy analysis (dbRDA) of correlations between immune-cell populations and microbial community composition at phylum and genus levels. phylum level (left), genus level (right), N, NOR group; CIA, CIA group. **(A)** SP. **(B)** PB. **(C)** MLN. **(D)** PPs. **(E)** IM. dbRDA1 and dbRDA2 represent the correlations of environmental variables with the first and second ordination axes, respectively. Arrow direction indicates positive association with the NOR or CIA group; arrow length reflects the magnitude of contribution.

Distance-based redundancy analysis (dbRDA) revealed significant separation in microbial community structure between groups under the constraint of OTU abundance environmental variables, with the dbRDA1 axis explaining the majority of the variation. Correlation analysis demonstrated significant associations between immune cell subsets and gut microbiota structure: T, B, and NK cells in SP (**Figure 8A**) were correlated with the gut microbiota at both the phylum and genus levels; NK and CD4^+^T cells in PB (**Figure 8B**) were correlated with bacterial community structure at the genus level; NK and CD8^+^T cells in MLN (**Figure 8C**) were correlated with the gut microbiota at the phylum and genus levels; T cells in PPs (**Figure 8D**) were correlated with bacterial communities at the genus level; and CD8^+^T cells in IM (**Figure 8E**) were correlated with gut microbiota structure at the phylum and genus levels. These results indicate pronounced tissue-specific associations between immune cell subsets and gut in CIA mice, with the most extensive correlations observed in the spleen. Furthermore, local CD8^+^T cells in the intestine are closely associated with microbial community structure and may play a key role in regulating intestinal immune balance in CIA mice.

#### 3.4.2 Association between α-diversity of gut microbiota and immune cell phenotype

**Figure 9:**
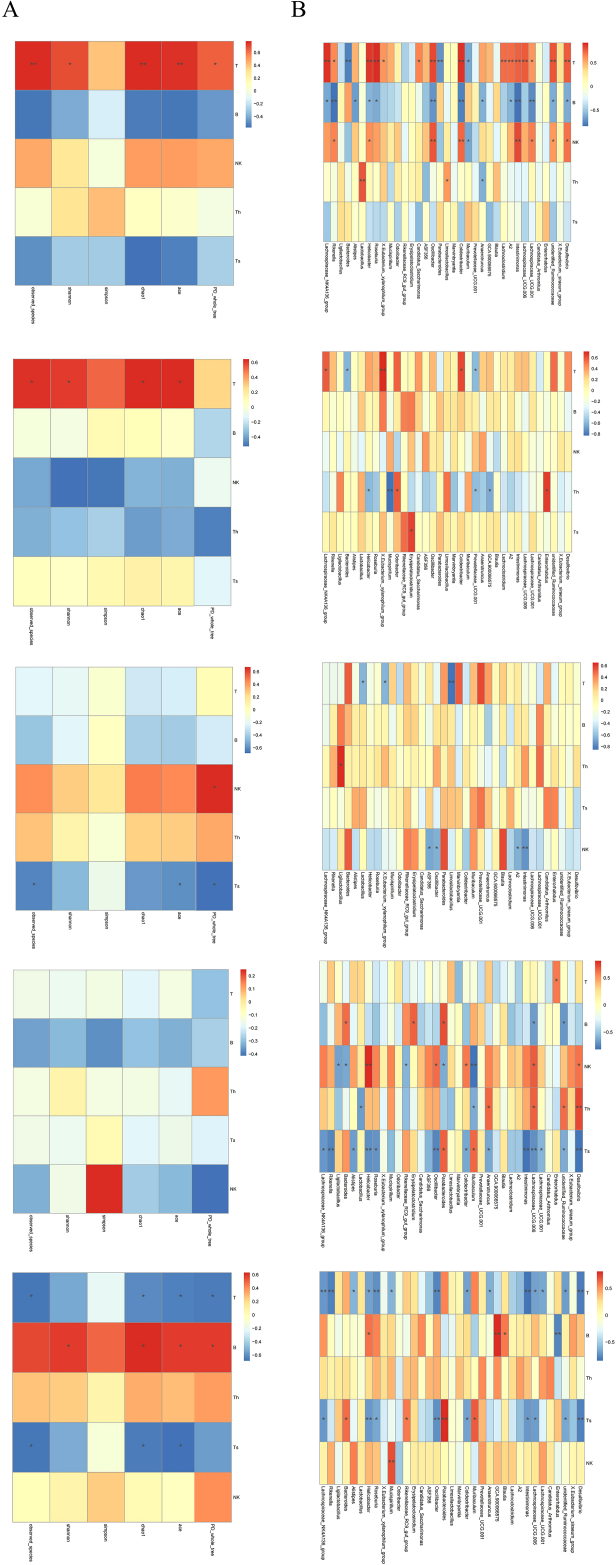
**(A)** Heatmap of correlations between α-diversity indices of the gut microbiota. **(B)** Heatmaps of correlations between T, B, NK, CD4^+^T, and CD8^+^T cells and the gut microbiota at the genus level. From top to bottom: SP, PB, MLN, PPs, and IM. Th = CD4^+^T cells; Ts = CD8^+^T cells. Red intensity indicates stronger positive correlations; blue intensity indicates stronger negative correlations (*P < 0.05, **P < 0.01).

Significant tissue-specific correlations were observed between gut α-diversity indices and immune cell levels. (**Figure 9A**) In CIA mice, increased α-diversity was positively correlated with T cell levels in the SP and PB, but negatively correlated with CD8^+^T cell levels in MLN and IM. These findings suggest that gut microbiota diversity may modulate systemic and local intestinal immune responses through distinct mechanisms.

#### 3.4.3 Correlation between specific bacteria and immune cell phenotype

Butyrate-producing bacteria *Lachnospiraceae_NK4A136_group* and *Roseburia* showed a positive correlation with T cell levels in the SP, a negative correlation with B cells, and a negative correlation with CD8^+^T cells in MLN and IM. In contrast, *Bacteroides* was negatively correlated with T cells in both SP and PB, positively correlated with B cells in MLN, negatively correlated with NK cells, and positively correlated with CD8^+^T cells in IM (**Figure 9B**) These results confirm that certain gut bacteria exhibit distinct tissue specificity in regulating both systemic and local immune responses in the host, with CD8^+^T cells potentially playing a central role in maintaining intestinal immune balance.

## 4 Discussion

Rheumatoid arthritis (RA) is difficult to treat due to its low remission rate and high disability rate. The disease staging model proposed by the European League Against Rheumatism (EULAR) reveals that the “pre-clinical stage” before the appearance of clinical arthritis is the key window for the development of RA (17, 18). The core of this stage lies in autoimmune disorders, which directly trigger subsequent inflammation and disease progression. Therefore, intervention in the pre-clinical stage, especially in the autoimmune link, is the key to breaking the situation and achieving disease blockade and deep remission.

The immunopathological mechanism of involves the combined action of multiple immune cells, and gut microbiota dysbiosis is an important factor driving the occurrence and development of autoimmune reactions. Compared with the NOR group, CIA mice showed a decrease in gut microbiota diversity and a significant change in the microbiota structure. The relative abundances of Firmicutes and Campylobacter were reduced, while the relative abundance of Bacteroidetes was increased, which is similar to the microbiota characteristics observed in RA patients. At the genus level, Parabacteroides and Muribaculum were significantly increased, while Lachnospiraceae_NK4A136_group and Roseburia were significantly decreased, and the latter was also often found to be reduced in RA patients (10). At the same time, the percentage of bacteria coated with IgA in the gut of CIA mice increased, and the SCFA content decreased significantly. SCFAs are key metabolites that can maintain the integrity of the intestinal barrier (12), and the production of different SCFAs is genus-specific: acetic acid can be produced by multiple microbiota, propionic acid mainly depends on specific genera such as Akkermansia, and butyric acid is highly concentrated in a few species such as Faecalibacterium prausnitzii and Eubacterium rectale (19). The correlation analysis in this study further verified the association between SCFAs and the microbiota structure. Microbiota function prediction Adaptive remodeling occurred in the functions of intestinal microbiota in CIA mice, such as multiple pathways, membrane protein expression, and enzyme composition and activity. The functional genes related to carbohydrate fermentation (especially xylan decomposition), amino acid metabolism, and drug efflux were significantly downregulated, while the pathways related to bacterial cell wall synthesis were significantly upregulated. It indicates that the intestinal microbiota of CIA mice has changed in composition, structure, and function.

In addition, the lymphoid subset phenotypes of CIA mice were altered both systemically and locally in the gut: T, B, and NK cells in the SP were all affected, and the local changes in the gut were particularly significant, manifested as a marked increase in CD8^+^T cells in the MLN and IM, and a trend of change was also observed in PPs; in contrast, the changes in PB lymphoid subsets were not significant, suggesting that the gut local area may be the initial site of immune activation, and its changes precede and may drive systemic immune disorders. Although the literature suggests that the gut microbiota can regulate immunity through DCs (20), this study found that there were no significant changes in the DC subsets of various immune tissues in CIA mice, suggesting that the immune imbalance caused by microbiota disorders in this model may not be mainly achieved through the antigen presentation pathway of DCs, and this mechanism needs to be further explored.

The literature shows that there is a complex bidirectional regulatory relationship between the gut microbiota and the host immune system: the microbiota not only dominates the development and function of local gut immunity, but can also affect the immune status of distal organs through pathways such as the gut-brain axis and gut-liver axis; at the same time, mucosal immunity also reciprocally shapes the microbiota composition (21–23). In this study, gut and systemic immune disorders were closely related to dysbiosis, suggesting that the two may influence each other. Among the effects of the microbiota on the immune system, the role of short-chain fatty acids (SCFAs) is particularly crucial. SCFAs can exert extensive and specific regulation on lymphocytes, not only affecting the activity of local gut lymphocytes but also acting on immune cells outside the gut (21, 22). This study found that both the proportion of gut CD8^+^ T cells and SCFA levels were significantly correlated with the microbiota structure, suggesting a possible functional link between the two. Notably, no significant association was found between the microbiota structure and CD4^+^ T cells in all local intestinal tissues. Previous studies have pointed out that the immune regulation of gut microbiota on CD4^+^ T cells usually requires dendritic cells (DCs) to present bacterial polysaccharides to activate the immune response (20). Further analysis of DC subsets in this study found that neither the percentage nor the activity of DC subsets in CIA mice changed significantly, which may be related to the lack of significant changes in CD4^+^ T cells. Previous studies have confirmed that RA patients generally have reduced SCFA levels, and higher levels of SCFAs may have a protective effect on high-risk individuals. Animal experiments also support the beneficial effects of SCFAs. In this study, as SCFAs decreased, the proportion of CD8^+^ T cells in the mesenteric lymph nodes and intestinal mucosa increased. SCFAs (especially butyric acid) have been shown to inhibit the production of inflammatory factors in T cells by enhancing histone acetylation and other means (24). Therefore, the observed decrease in SCFA levels in this study may have weakened this inhibitory regulation, leading to the overactivation and proliferation of local CD8^+^ T cells in the gut.

As key cytotoxic lymphocytes, CD8^+^ T cells are responsible for clearing infected and tumor cells in normal immunity. However, in patients with RA, they show abnormal activation and dysfunction, can recognize autoantigens such as citrullinated fibrinogen, and induce chondrocyte apoptosis and bone erosion by releasing perforin and granzyme (4). In the early stage of CIA, CD8^+^ T cells may participate in the inflammatory response and cause joint damage through cytotoxic effects and cytokine production (such as interferon γ) (25). The proportion of CD8^+^ T cells in peripheral blood monocytes and spleens of CIA rats increases, accompanied by a decrease in CD4^+^ T cells, further indicating that CD8^+^ T cells play an important role in activating the immune response and regulating CIA inflammation (26, 27). In addition, besides the quantitative changes, the functional heterogeneity of CD8^+^ T cells may also be the key to linking SCFAs and inflammation. It is known that CD8^+^ T cells can be divided into subsets with different functions: including short-term effector subsets with high migration, strong inflammation and cytotoxicity; effector memory subsets that settle in the periphery and have anti-apoptotic ability; central memory subsets that can proliferate rapidly but have low cytotoxicity; and inhibitory subsets that inhibit inflammation by secreting IL-10. These subsets show obvious heterogeneous expression and functional differences in RA (28). Therefore, subsequent studies will focus on exploring the specific associations between each subset of CD8^+^ T cells and gut microbiota and their metabolite SCFAs.

This study also found that the percentage of splenic B cells increased significantly after modeling, accompanied by an increase in the level of serum Type II Collagen IgG Antibody, suggesting that the changes in splenic B cells may be related to short-chain fatty acids (SCFAs) entering the bloodstream through the gut-liver axis and are related to affecting B cells. However, the levels of SCFAs in feces do not directly reflect the local SCFAs concentration in the spleen (29, 30). Although it was observed that the decrease in fecal SCFAs levels coexisted with the increase in splenic B cells after modeling, this phenomenon was inconsistent with the generally reported conclusion in the literature that SCFAs can promote the differentiation of B cells into antibody-producing cells (31). In addition, correlation analyses of the proportions of B cells in multiple sites such as the spleen, peripheral blood, mesenteric lymph nodes, Peyer’s patches, and intestinal mucosa, as well as fecal IgA levels, did not find significant associations. Based on the above results, we speculate that the increase in splenic B cells has no direct connection with fecal IgA levels, but its changes show a good correlation with the intestinal microbial composition, suggesting that the increase in splenic B cells may be somewhat associated with changes in the gut microbiota.

The research revealed a co-variation relationship among gut microbiota, immune cells, and metabolites in the CIA model through correlation analysis, providing clues for understanding the “gut-immune” axis of arthritis. However, these associations only indicate co-variation among variables and cannot establish causal relationships. In addition, although the pathway of “microbiota-SCFAs-immune cells” was speculated, correlation analysis cannot exclude the confounding effects of other unmeasured factors. Therefore, this causal mechanism remains a reasonable hypothesis at present and needs to be further verified through experiments such as microbial transplantation, SCFA supplementation, or immune cell intervention.

In the aspect of functional prediction analysis, changes in the gut microbiota of CIA mice in metabolic and ecological functions were found, which were highly consistent with the decrease in butyrate-producing bacteria (such as Lachnospiraceae_NK4A136_group and Roseburia) observed at the taxonomic level. These bacteria are key executors of the fermentation of dietary fibers (such as xylan), and their reduction may directly lead to a decline in the ability to synthesize SCFAs, which may be potentially related to the abnormal expansion of CD8^+^ T cells in the intestine, and may further accelerate the progression of arthritis. This functional prediction is based on the functional prediction of 16S rDNA sequencing, and its results are only inferences of functional potential, unable to reflect the functional differences between strains and the actual expression status of genes, nor can it detect the true metabolite levels, and experimental verification needs to be combined with multi-omics technologies.

In summary, we detected the gut microbiota and immune cell phenotypes of CIA mice by techniques such as 16S rDNA sequencing, SCFAs metabolomics, and flow cytometry, and performed cross-omics network analysis. The research results confirmed that the gut microbiota of CIA mice was significantly disordered, the synthesis level of SCFAs decreased, and there was a potential association with the expansion of intestinal local CD8^+^ T cells, resulting in the imbalance of T/B cells in the SP and ultimately leading to the occurrence and development of arthritis. Based on these findings, future exploration of RA treatment can consider focusing on restoring the balance of gut microbiota and regulating the functions of CD8^+^ T cells and B cells, providing new ideas for the treatment of RA.

## 5 Conclusion

In CIA mice, the characteristics of gut microbiota dysregulation during the development of arthritis were the decrease of Lachnospiraceae_NK4A136_group and Roseburia, the increase of Bacteroidota, the decrease of Firmicutes, and the significant decrease of SCFAs. These changes may lead to a significant increase in CD8^+^ T cells in the MLN and IM, a decrease in T cells in the SP, and an increase in B cells. Cross-group analysis further showed that intestinal imbalance in CIA mice may reduce the synthesis of SCFAs; this decrease may be related to the expansion of intestinal local CD8^+^ T cells, promote the activation of splenic B cells, and ultimately contribute to the progression of arthritis.

## Data Availability

The data presented in the study are deposited in the NCBI repository, accession number PRJNA1355038.
