# Supplementary material for: CD8+ T cells mediate the impact of gut dysbiosis and short-chain fatty acid deficiency on accelerated arthritis progression in collagen-induced arthritis mice
Source: Front Immunol. 2025 Nov 14;16:1702792. doi: 10.3389/fimmu.2025.1702792 (PMC12660273; doi:10.3389/fimmu.2025.1702792)
Supplement: Supplementary file 1 [file DataSheet1.docx]

**1.Supplementary Data**

**Anesthesia, euthanasia, and handling of mice**

In this experiment, inhalation anesthesia was performed on mice using a small animal anesthesia machine (Shenzhen RWD TAIJI Series), with the experimental anesthetic being Isoflurane (Batch No.: 2024081901).The inflammatory peak occurred approximately 10 weeks after the initial immunization and all mice were anesthetized and euthanized at the 11th week after the initial immunization.

Install the drug adapter: Align and insert it into the snap-fit mechanism, then tighten by rotating. Remove the sealing cap and ensure the sealing ring is aligned with the "0" mark. Align the adapter with the anesthetic bottle’s port, insert, and tighten.Open the oxygen source (not exceeding 0.42 MPa), turn the oxygen flow adjustment knob counterclockwise and observe the position of the float in the flowmeter to adjust the flow rate to 1.0 L/min.

Place the mouse into the anesthesia induction chamber. Turn the outlet switch of the induction chamber to the right, toward the "ON" indicator direction, to allow anesthetic gas to flow into the induction chamber. Press the dial lock button and rotate the vaporizer dial to adjust the anesthetic concentration to an appropriate level (3.0–4.0%).

After induction anesthesia is complete, rotate the vaporizer dial to adjust the concentration to a suitable level for euthanasia (5.0%). Turn the outlet switch of the anesthesia mask to the right, toward the "ON" indicator direction, to allow anesthetic gas to flow into the mask. Remove the mouse from the induction chamber and position its nose inside the anesthesia mask. Confirm the mouse's death approximately 5 minutes after anesthesia, immediately position the mouse in a supine position and secure it. Using a 1-mL syringe, insert the needle at an appropriate angle below the sternum or xiphoid process. After entering the ventricle, gently withdraw the plunger to collect blood. Subsequently, under sterile conditions, collect fecal samples, cecal contents, spleen, intestinal tract, right hind limb, and other tissues and organs for further research.

1. **Supplementary Tables**

**Supplementary Table 1：**Dynamic changes of the arthritis index (AI) in mouse joints

|  | NOR (Mean±SD) | CIA (Mean±SD) |
| --- | --- | --- |
| 4w | 0 | 3.83±0.75 |
| 5w | 0 | 4.67±0.52 |
| 6w | 0 | 5.67±0.52 |
| 7w | 0 | 7.33±0.52 |
| 8w | 0 | 11.00±0.89 |
| 9w | 0 | 11.67±0.52 |
| 10w | 0 | 12.00±0.00 |
| 11w | 0 | 11.17±0.41 |

**Supplementary Table 2A**：immune cells in peripheral blood (PB)

|  | NOR (Mean±SD) | CIA (Mean±SD) | t-value | P-value |
| --- | --- | --- | --- | --- |
| T | 30.13 ± 3.96 | 24.41 ± 7.88 | 1.588 | 0.143 |
| B | 57.55 ± 12.06 | 57.22 ± 17.73 | 0.037 | 0.971 |
| NK | 8.43 ± 2.12 | 8.93 ± 2.42 | -0.382 | 0.71 |
| CD4+T | 56.22 ± 4.57 | 60.77 ± 6.62 | -1.385 | 0.196 |
| CD8+T | 31.65 ± 5.17 | 28.65 ± 4.30 | 1.093 | 0.3 |

**Supplementary Table 2B**：immune cells in the spleen (SP)

|  | NOR (Mean±SD) | CIA (Mean±SD) | t-value | P-value |
| --- | --- | --- | --- | --- |
| T | 43.00 ± 3.24 | 16.67 ± 3.37 | 13.811 | ＜0.001* |
| B | 42.64 ± 6.55 | 71.16 ± 2.88 | -9.766 | ＜0.001* |
| NK | 3.87 ± 0.91 | 1.15 ± 0.20 | 7.185 | ＜0.001* |
| CD4+T | 63.44 ± 3.86 | 62.25 ± 3.28 | 0.571 | 0.58 |
| CD8+T | 24.17 ± 4.13 | 27.24 ± 6.33 | -0.996 | 0.343 |

**Supplementary Table 2C**：Dendritic cells and their subsets in peripheral blood (PB)

|  | NOR (Mean±SD) | CIA (Mean±SD) | t-value | P-value |
| --- | --- | --- | --- | --- |
| cDC | 58.15 ± 9.44 | 55.28 ± 18.77 | 0.335 | 0.745 |
| pDC | 38.16 ± 5.02 | 45.07 ± 21.63 | -0.763 | 0.463 |
| CD8α+cDC1 | 0.59 ± 0.14 | 1.14 ± 0.69 | -1.902 | 0.111 |
| CD103+cDC1 | 0.78 ± 0.79 | 3.45 ± 6.79 | -0.957 | 0.361 |
| CD11b+cDC2 | 72.58 ± 8.65 | 64.18 ± 11.96 | 1.394 | 0.194 |

**Supplementary Table 2D**:Dendritic cells and their subsets in the spleen (SP)

|  | NOR (Mean±SD) | CIA (Mean±SD) | t-value | P-value |
| --- | --- | --- | --- | --- |
| cDC | 82.11±5.54 | 82.33±6.07 | -0.066 | 0.949 |
| pDC | 14.99±2.45 | 17.58±5.28 | -1.086 | 0.303 |
| CD8α+cDC1 | 20.06±4.07 | 17.00±3.70 | 1.36 | 0.204 |
| CD103+cDC1 | 4.17 ± 2.034 | 7.37 ± 5.914 | -1.256 | 0.238 |
| CD11b+cDC2 | 17.35±4.85 | 20.33±5.45 | -0.998 | 0.342 |
| CD8α+cDC1/CD11b+cDC2 | 1.28±0.62 | 0.87±0.21 | 1.52 | 0.159 |

**Supplementary Table 3A**：immune cells in the mesenteric lymph nodes (MLN)

|  | NOR (Mean±SD) | CIA (Mean±SD) | t-value | P-value |
| --- | --- | --- | --- | --- |
| T | 53.62 ± 3.45 | 54.45 ± 5.92 | -0.297 | 0.773 |
| B | 31.50 ± 3.71 | 33.09 ± 2.87 | -0.832 | 0.425 |
| NK | 1.37 ± 0.47 | 0.63 ± 0.32 | 3.188 | 0.01* |
| CD4+T | 72.46 ± 12.82 | 65.77 ± 12.67 | 0.908 | 0.385 |
| CD8+T | 17.26 ± 5.40 | 36.88 ± 8.58 | -4.742 | 0.001* |

**Supplementary Table 3B**：immune cells in Peyer's patches (PPs)

|  | NOR (Mean±SD) | CIA (Mean±SD) | t-value | P-value |
| --- | --- | --- | --- | --- |
| T | 27.79 ± 2.55 | 29.87 ± 3.47 | -1.184 | 0.264 |
| B | 59.34 ± 5.51 | 61.10 ± 7.80 | -0.452 | 0.661 |
| NK | 0.65 ± 0.22 | 0.90 ± 0.23 | -1.897 | 0.087 |
| CD4+T | 67.98 ± 5.99 | 64.74 ± 10.96 | 0.637 | 0.538 |
| CD8+T | 15.81 ± 2.87 | 17.81 ± 3.31 | -1.112 | 0.292 |

**Supplementary Table 3C**：immune cells in the intestinal mucosa (IM)

|  | NOR (Mean±SD) | CIA (Mean±SD) | t-value | P-value |
| --- | --- | --- | --- | --- |
| T | 17.91 ± 3.01 | 68.48 ± 10.58 | -11.258 | ＜0.001* |
| B | 1.26 ± 0.58 | 0.75 ± 0.24 | 2 | 0.088 |
| NK | 0.41 ± 0.25 | 0.32 ± 0.21 | 0.646 | 0.533 |
| CD4+T | 11.67 ± 2.28 | 10.02 ± 2.90 | 1.1 | 0.297 |
| CD8+T | 57.72±12.72 | 79.53±4.41 | -3.967 | 0.007* |

**Supplementary Table 4A**：Dendritic cells and their subsets in mesenteric lymph nodes (MLN)

|  | NOR (Mean±SD) | CIA (Mean±SD) | t-value | P-value |
| --- | --- | --- | --- | --- |
| cDC | 92.54±2.23 | 94.77±2.82 | -1.523 | 0.159 |
| pDC | 4.28±2.72 | 4.18±2.37 | 0.067 | 0.948 |
| CD8α+cDC1 | 5.84±7.36 | 3.58±4.34 | 0.648 | 0.531 |
| CD103+cDC1 | 37.00±24.95 | 40.43±27.03 | -0.228 | 0.824 |
| CD11b+cDC2 | 24.65±12.83 | 30.90±6.66 | -0.23 | 0.314 |

**Supplementary Table 4B**：Dendritic cells and their subsets in Peyer's patches (PPs)

|  | NOR (Mean±SD) | CIA (Mean±SD) | t-value | P-value |
| --- | --- | --- | --- | --- |
| cDC | 86.06±4.09 | 90.75±4.10 | -1.984 | 0.075 |
| pDC | 5.64±3.71 | 4.29±3.49 | 0.653 | 0.529 |
| CD8α+cDC1 | 2.33±1.35 | 1.36±1.50 | 1.166 | 0.271 |
| CD103+cDC1 | 13.64±5.01 | 18.15±4.68 | -1.613 | 0.138 |
| CD11b+cDC2 | 49.89±29.33 | 65.32±5.43 | -1.267 | 0.257 |

**Supplementary Table 4C**:Dendritic cells and their subsets in the intestinal mucosa (IM)

|  | NOR (Mean±SD) | CIA (Mean±SD) | t-value | P-value |
| --- | --- | --- | --- | --- |
| cDC | 87.37±22.65 | 97.82±1.03 | -1.13 | 0.31 |
| pDC | 0.43±0.40 | 1.07±1.08 | -1.352 | 0.206 |
| CD8α+cDC1 | 2.09±1.36 | 0.82±0.69 | 2.038 | 0.079 |
| CD103+cDC1 | 4.31±3.36 | 0.87±0.88 | 2.424 | 0.054 |
| cDC2 | 5.99±3.75 | 3.14±1.39 | 1.744 | 0.129 |

**Supplementary Table 5**：SCFA and coated IgA bacteria levels in mice feces (μg/mL)

|  | NOR (Mean±SD) | CIA (Mean±SD) | t-value | P-value |
| --- | --- | --- | --- | --- |
| IgA(%) | 29.9±12.1 | 44.29±6.78 | -2.521 | 0.03* |
| AA | 863.22±128.96 | 666.95±123.01 | 2.697 | 0.022* |
| PA | 102.45±28.24 | 71.96±35.65 | 1.642 | 0.132 |
| IBA | 7.52±3.54 | 2.43±0.81 | 3.438 | 0.016* |
| BA | 240.03±35.72 | 180.03±80.70 | 1.665 | 0.127 |
| IVA | 5.07±1.65 | 3.02±0.35 | 2.975 | 0.014* |
| VA | 8.21±4.60 | 7.69±3.84 | 0.214 | 0.835 |

**Supplementary Table 6**：Correlation Analysis of B Cells and IgA-Coated Bacteria in the CIA Group

|  |  |  | B cells | IgA |
| --- | --- | --- | --- | --- |
| PB | B cells | Pearson correlation | 1 | -0.598 |
|  |  | Significance (two-tailed) |  | .210 |
| SP | B cells | Pearson correlation | 1 | 0.448 |
|  |  | Significance (two-tailed) |  | 0.373 |
| MLN | B cells | Pearson correlation | 1 | -0.327 |
|  |  | Significance (two-tailed) |  | 0.527 |
| PPs | B cells | Pearson correlation | 1 | -0.122 |
|  |  | Significance (two-tailed) |  | 0.818 |
| IM | B cells | Pearson correlation | 1 | 0.516 |
|  |  | Significance (two-tailed) |  | 0.295 |
|  |  | Number of cases | 6 | 6 |
